# (2*E*)-1-(Pyridin-2-yl)-3-(2,4,5-tri­meth­oxy­phen­yl)prop-2-en-1-one

**DOI:** 10.1107/S1600536813015821

**Published:** 2013-06-12

**Authors:** Suchada Chantrapromma, Thitipone Suwunwong, Nawong Boonnak, Hoong-Kun Fun

**Affiliations:** a Department of Chemistry, Faculty of Science, Prince of Songkla University, Hat-Yai, Songkhla 90112, Thailand; b Faculty of Traditional Thai Medicine, Prince of Songkla University, Hat-Yai, Songkhla 90112, Thailand; c X-ray Crystallography Unit, School of Physics, Universiti Sains Malaysia, 11800 USM, Penang, Malaysia; d Department of Pharmaceutical Chemistry, College of Pharmacy, King Saud University, PO Box 2457, Riyadh 11451, Saudi Arabia

## Abstract

The title heteroaryl chalcone derivative, C_17_H_17_NO_4_, is close to planar: the dihedral angle between the pyridine and benzene rings is 3.71 (11)° and the meth­oxy C atoms deviate from their attached ring by 0.046 (3), −0.044 (2) and 0.127 (3) Å. The disposition of the pyridine N atom and the carbonyl group is *anti* [N—C—C—O = −177.7 (2)°]. In the crystal, mol­ecules are linked by weak C—H⋯N and C—H⋯O inter­actions into (100) sheets and an aromatic π–π stacking inter­action between the pyridine and benzene ring, with a centroid–centroid separation of 3.7036 (14) Å also occurs.

## Related literature

For the fluorescence properties of heteroaryl chalcones, see: Suwunwong *et al.* (2011 ▶). For related structures, see: Chantrapromma *et al.* (2009 ▶); Fun *et al.* (2010 ▶, 2011 ▶); Suwunwong *et al.* (2012 ▶). For the stability of the temperature controller used in the data collection, see: Cosier & Glazer (1986 ▶).
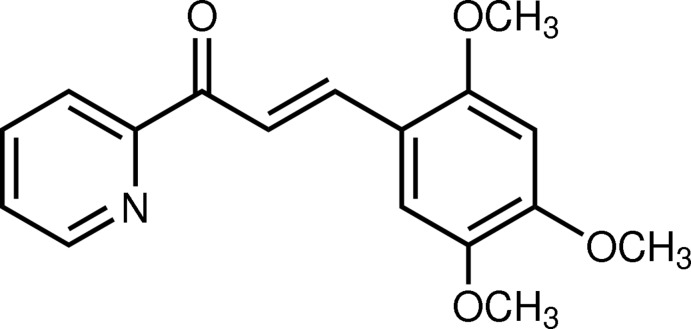


## Experimental

### 

#### Crystal data

C_17_H_17_NO_4_
*M* *_r_* = 299.32
Monoclinic, 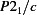

*a* = 8.4047 (3) Å
*b* = 8.7285 (3) Å
*c* = 19.5086 (7) Å
β = 94.113 (2)°
*V* = 1427.47 (9) Å^3^
*Z* = 4
Mo *K*α radiation
μ = 0.10 mm^−1^
*T* = 100 K
0.32 × 0.27 × 0.16 mm

#### Data collection

Bruker APEXII CCD diffractometer
Absorption correction: multi-scan (*SADABS*; Bruker, 2005 ▶) *T*_min_ = 0.969, *T*_max_ = 0.984
14970 measured reflections
3792 independent reflections
2435 reflections with *I* > 2σ(*I*)
*R*_int_ = 0.054

#### Refinement

*R*[*F* ^2^ > 2σ(*F* ^2^)] = 0.067
*wR*(*F* ^2^) = 0.188
*S* = 1.07
3792 reflections
202 parameters
H-atom parameters constrained
Δρ_max_ = 0.58 e Å^−3^
Δρ_min_ = −0.29 e Å^−3^

### 

Data collection: *APEX2* (Bruker, 2005 ▶); cell refinement: *SAINT* (Bruker, 2005 ▶); data reduction: *SAINT*; program(s) used to solve structure: *SHELXTL* (Sheldrick, 2008 ▶); program(s) used to refine structure: *SHELXTL* ; molecular graphics: *SHELXTL*; software used to prepare material for publication: *SHELXTL*, *PLATON* (Spek, 2009 ▶), *Mercury* (Macrae *et al.*, 2006 ▶) and *publCIF* (Westrip, 2010 ▶).

## Supplementary Material

Crystal structure: contains datablock(s) global, I. DOI: 10.1107/S1600536813015821/hb7090sup1.cif

Structure factors: contains datablock(s) I. DOI: 10.1107/S1600536813015821/hb7090Isup2.hkl

Supplementary material file. DOI: 10.1107/S1600536813015821/hb7090Isup3.cml

Additional supplementary materials:  crystallographic information; 3D view; checkCIF report

## Figures and Tables

**Table 1:** Hydrogen-bond geometry (Å, °)

*D*—H⋯*A*	*D*—H	H⋯*A*	*D*⋯*A*	*D*—H⋯*A*
C3—H3*A*⋯O4^i^	0.93	2.39	3.277 (3)	160
C15—H15*A*⋯N1^ii^	0.96	2.47	3.349 (3)	153

Symmetry codes: (i) 
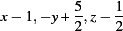
; (ii) 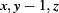.

**Fig. 1.**
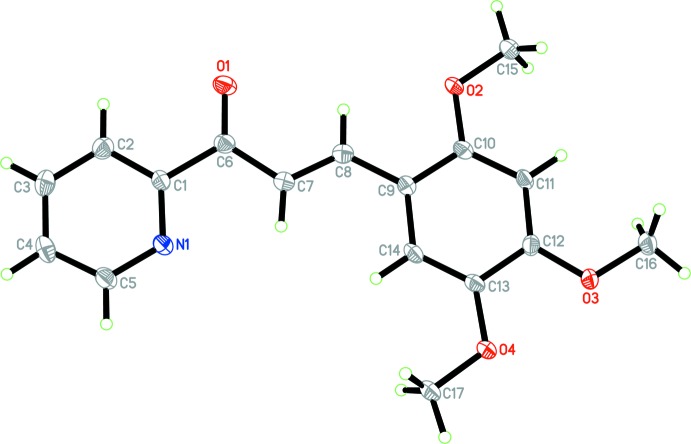
The molecular structure of (I), showing 50% probability displacement ellipsoids.

**Fig. 2.**
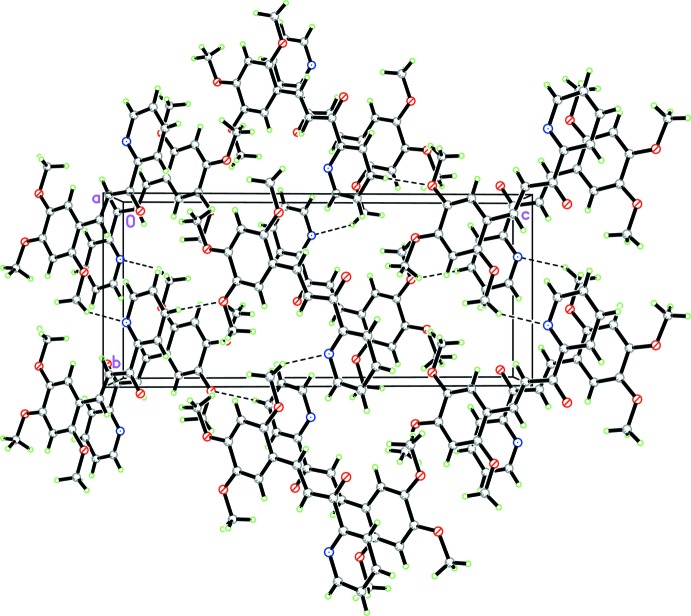
The crystal packing of (I) viewed along the a axis, showing molecular sheets parallel to the bc plane. C—H···O and C—H···N interactions are shown as dashed lines.

**Fig. 3.**
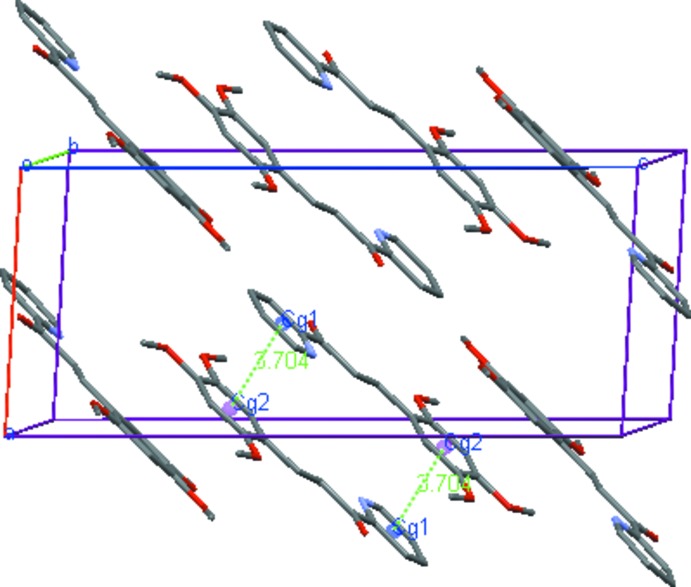
The crystal packing of (I) viewed approximately along the b axis, showing π–π interactions; Cg1 and Cg2 are the centroids of N1/C1–C5 and C9–C14 rings, respectively. H atoms were omitted for clarity.

## supplementary crystallographic information

### Comment

As part of our ongoing studies of the crystal structures and fluorescent properties of chalcones and heteroaryl chalcones (Chantrapromma *et al.*, 2009; Fun *et al.*, 2010, 2011; Suwunwong *et al.*, 2011, 2012), the title heteroaryl chalcone derivative (I) was synthesized and studied for fluorescent property and for further cyclization purpose. It was also screened for antibacterial and antityrosinase activities and found to be inactive. However it exhibited fluorescence with the emission wavelength at 530 nm when excited at 401 nm. Herein we report the crystal structure of (I).

The molecule of the title heteroaryl chalcone (Fig. 1) exists in an *E* conformation with respect to the C7═C8 double bond [1.343 (3) Å] and the torsion angle C6–C7–C8–C9 = 179.4 (2)°. The molecule is close to planar with the dihedral angle between pyridine and benzene rings being 3.71 (11)°. Atoms of the middle propenone bridge (C6, C7, C8 and O1) lie almost on the same plane as indicated by the torsion angle O1–C6–C7–C8 = -1.3 (4)°. The mean plane through this bridge makes dihedral angles of 3.03 (15) and 3.06 (15)° with the planes of pyridine and benzene rings, respectively. All the three substituted methoxy groups of 2,4,5-trimethoxyphenyl unit are close to co-planar with the bound benzene ring with the *r.m.s*. deviation of 0.0290 (2) Å for the twelve non H atoms and the torsion angles C15–O2–C10–C11 = 0.4 (3)°, C16–O3–C12–C13 = 177.9 (2)° and C17–O4–C13–C14 = -2.6 (3)°. The bond distances are comparable with those in related structures (Chantrapromma *et al.*, 2009; Fun *et al.*, 2010; 2011 and Suwunwong *et al.*, 2012).

In the crystal (Fig. 2), only one methoxy group (at atom C10) is involved in weak C—H···N and C—H···O interactions (Table 1). The molecules are linked by weak C15—H15A···N1 and C3—H3A···O4 interactions (Table 1) into (100) sheets. π–π interaction with the *Cg*_1_···*Cg*_2_^iii^ distance of 3.7036 (14) Å (iii = 2 - *x*, 2 - *y*, -*z*) was presented (Fig. 3); *Cg*_1_ and *Cg*_2_ are the centroids of N1/C1–C5 and C9–C14 rings, respectively.

### Experimental

The title compound was synthesized by the condensation of 2,4,5-trimethoxybenzaldehyde (0.40 g, 2 mmol) with 2-acetylpyridine (0.20 g, 2 mmol) in ethanol (30 ml) in the presence of 30% NaOH(aq) (5 ml). After stirring in ice bath at 278 K for 4 h, the resulting yellow solid appeared and was then collected by filtration, washed with distilled water, dried and purified by repeated recrystallization from acetone. Yellow blocks of (I) were recrystallized from acetone solution by the slow evaporation of the solvent at room temperature after several days, Mp. 428–429 K.

### Refinement

All H atoms were positioned geometrically and allowed to ride on their parent atoms, with d(C—H) = 0.93 Å for aromatic and CH, and 0.96 Å for CH_3_ atoms. The *U*_iso_ values were constrained to be 1.5*U*_eq_ of the carrier atom for methyl H atoms and 1.2*U*_eq_ for the remaining H atoms. A rotating group model was used for the methyl groups.

### Crystal data

**Table:**

C_17_H_17_NO_4_	*F*(000) = 632
*M**_r_* = 299.32	*D*_x_ = 1.393 Mg m^−^^3^
Monoclinic, *P*2_1_/*c*	Melting point = 428–429 K
Hall symbol: -P 2ybc	Mo *K*α radiation, λ = 0.71073 Å
*a* = 8.4047 (3) Å	Cell parameters from 3792 reflections
*b* = 8.7285 (3) Å	θ = 2.1–29.0°
*c* = 19.5086 (7) Å	µ = 0.10 mm^−^^1^
β = 94.113 (2)°	*T* = 100 K
*V* = 1427.47 (9) Å^3^	Block, yellow
*Z* = 4	0.32 × 0.27 × 0.16 mm

### Data collection

**Table:**

Bruker APEXII CCD diffractometer	3792 independent reflections
Radiation source: sealed tube	2435 reflections with *I* > 2σ(*I*)
Graphite monochromator	*R*_int_ = 0.054
φ and ω scans	θ_max_ = 29.0°, θ_min_ = 2.1°
Absorption correction: multi-scan (*SADABS*; Bruker, 2005)	*h* = −10→11
*T*_min_ = 0.969, *T*_max_ = 0.984	*k* = −11→11
14970 measured reflections	*l* = −26→26

### Refinement

**Table:**

Refinement on *F*^2^	Primary atom site location: structure-invariant direct methods
Least-squares matrix: full	Secondary atom site location: difference Fourier map
*R*[*F*^2^ > 2σ(*F*^2^)] = 0.067	Hydrogen site location: inferred from neighbouring sites
*wR*(*F*^2^) = 0.188	H-atom parameters constrained
*S* = 1.07	*w* = 1/[σ^2^(*F*_o_^2^) + (0.0802*P*)^2^ + 1.2643*P*] where *P* = (*F*_o_^2^ + 2*F*_c_^2^)/3
3792 reflections	(Δ/σ)_max_ = 0.001
202 parameters	Δρ_max_ = 0.58 e Å^−^^3^
0 restraints	Δρ_min_ = −0.29 e Å^−^^3^

### Special details

**Table:**

Experimental. The crystal was placed in the cold stream of an Oxford Cryosystems Cobra open-flow nitrogen cryostat (Cosier & Glazer, 1986) operating at 100.0 (1) K.
Geometry. All e.s.d.'s (except the e.s.d. in the dihedral angle between two l.s. planes) are estimated using the full covariance matrix. The cell e.s.d.'s are taken into account individually in the estimation of e.s.d.'s in distances, angles and torsion angles; correlations between e.s.d.'s in cell parameters are only used when they are defined by crystal symmetry. An approximate (isotropic) treatment of cell e.s.d.'s is used for estimating e.s.d.'s involving l.s. planes.
Refinement. Refinement of *F*^2^ against ALL reflections. The weighted *R*-factor *wR* and goodness of fit *S* are based on *F*^2^, conventional *R*-factors *R* are based on *F*, with *F* set to zero for negative *F*^2^. The threshold expression of *F*^2^ > σ(*F*^2^) is used only for calculating *R*-factors(gt) *etc*. and is not relevant to the choice of reflections for refinement. *R*-factors based on *F*^2^ are statistically about twice as large as those based on *F*, and *R*- factors based on ALL data will be even larger.

### Fractional atomic coordinates and isotropic or equivalent isotropic displacement parameters (Å^2^)

**Table:**

	*x*	*y*	*z*	*U*_iso_*/*U*_eq_	
O1	0.6126 (2)	0.93348 (19)	−0.05940 (8)	0.0229 (4)	
O2	0.9130 (2)	0.60091 (19)	0.09428 (8)	0.0234 (4)	
O3	1.3110 (2)	0.78070 (19)	0.26882 (8)	0.0219 (4)	
O4	1.2622 (2)	1.05914 (19)	0.22949 (8)	0.0234 (4)	
N1	0.7107 (2)	1.3210 (2)	−0.02122 (10)	0.0194 (4)	
C1	0.6314 (3)	1.2048 (3)	−0.05320 (11)	0.0166 (5)	
C2	0.5113 (3)	1.2269 (3)	−0.10560 (12)	0.0221 (5)	
H2A	0.4597	1.1436	−0.1270	0.027*	
C3	0.4706 (3)	1.3755 (3)	−0.12499 (12)	0.0239 (5)	
H3A	0.3901	1.3938	−0.1592	0.029*	
C4	0.5517 (3)	1.4957 (3)	−0.09262 (12)	0.0234 (5)	
H4A	0.5276	1.5964	−0.1050	0.028*	
C5	0.6702 (3)	1.4634 (3)	−0.04114 (12)	0.0222 (5)	
H5A	0.7241	1.5450	−0.0194	0.027*	
C6	0.6774 (3)	1.0442 (3)	−0.03057 (11)	0.0184 (5)	
C7	0.7993 (3)	1.0306 (3)	0.02671 (11)	0.0214 (5)	
H7A	0.8459	1.1188	0.0459	0.026*	
C8	0.8451 (3)	0.8932 (3)	0.05195 (11)	0.0199 (5)	
H8A	0.7949	0.8080	0.0315	0.024*	
C9	0.9661 (3)	0.8631 (3)	0.10836 (11)	0.0178 (5)	
C10	0.9984 (3)	0.7123 (3)	0.12964 (11)	0.0172 (5)	
C11	1.1109 (3)	0.6809 (3)	0.18425 (11)	0.0165 (5)	
H11A	1.1281	0.5805	0.1989	0.020*	
C12	1.1961 (3)	0.7987 (3)	0.21634 (11)	0.0173 (5)	
C13	1.1689 (3)	0.9518 (3)	0.19514 (11)	0.0176 (5)	
C14	1.0550 (3)	0.9814 (3)	0.14248 (11)	0.0177 (5)	
H14A	1.0361	1.0824	0.1290	0.021*	
C15	0.9402 (3)	0.4457 (3)	0.11480 (13)	0.0268 (6)	
H15A	0.8753	0.3791	0.0853	0.040*	
H15B	0.9130	0.4327	0.1614	0.040*	
H15C	1.0507	0.4207	0.1115	0.040*	
C16	1.3405 (3)	0.6280 (3)	0.29434 (12)	0.0230 (5)	
H16A	1.4170	0.6315	0.3333	0.035*	
H16B	1.3814	0.5662	0.2589	0.035*	
H16C	1.2427	0.5844	0.3079	0.035*	
C17	1.2442 (3)	1.2141 (3)	0.20699 (13)	0.0248 (5)	
H17A	1.3223	1.2770	0.2319	0.037*	
H17B	1.1392	1.2497	0.2153	0.037*	
H17C	1.2590	1.2199	0.1587	0.037*	

### Atomic displacement parameters (Å^2^)

**Table:**

	*U*^11^	*U*^22^	*U*^33^	*U*^12^	*U*^13^	*U*^23^
O1	0.0286 (9)	0.0155 (9)	0.0246 (8)	−0.0034 (7)	0.0011 (7)	−0.0018 (7)
O2	0.0307 (10)	0.0092 (8)	0.0283 (8)	−0.0006 (7)	−0.0102 (7)	0.0001 (7)
O3	0.0235 (9)	0.0135 (8)	0.0271 (8)	0.0002 (7)	−0.0089 (7)	0.0034 (7)
O4	0.0269 (9)	0.0110 (9)	0.0307 (9)	−0.0018 (7)	−0.0094 (7)	0.0017 (7)
N1	0.0240 (10)	0.0142 (10)	0.0199 (9)	0.0012 (8)	0.0006 (8)	0.0004 (8)
C1	0.0188 (11)	0.0145 (11)	0.0168 (10)	0.0014 (9)	0.0030 (8)	0.0013 (9)
C2	0.0211 (12)	0.0231 (13)	0.0217 (11)	−0.0010 (10)	−0.0015 (9)	0.0009 (10)
C3	0.0214 (12)	0.0285 (14)	0.0216 (11)	0.0053 (11)	−0.0002 (9)	0.0035 (10)
C4	0.0250 (13)	0.0195 (13)	0.0263 (12)	0.0072 (10)	0.0063 (10)	0.0062 (10)
C5	0.0268 (13)	0.0155 (12)	0.0242 (11)	0.0022 (10)	0.0021 (9)	−0.0002 (9)
C6	0.0224 (12)	0.0167 (12)	0.0163 (10)	−0.0011 (10)	0.0033 (8)	−0.0007 (9)
C7	0.0249 (12)	0.0162 (12)	0.0225 (11)	−0.0009 (10)	−0.0031 (9)	−0.0013 (9)
C8	0.0206 (11)	0.0176 (12)	0.0214 (10)	−0.0014 (10)	−0.0007 (9)	−0.0009 (9)
C9	0.0210 (12)	0.0139 (11)	0.0183 (10)	0.0005 (9)	−0.0003 (9)	0.0011 (9)
C10	0.0189 (11)	0.0125 (11)	0.0202 (10)	−0.0009 (9)	0.0020 (9)	0.0005 (9)
C11	0.0167 (11)	0.0112 (11)	0.0216 (10)	0.0006 (9)	0.0008 (8)	0.0029 (9)
C12	0.0158 (11)	0.0172 (12)	0.0186 (10)	0.0023 (9)	−0.0014 (8)	0.0024 (9)
C13	0.0200 (11)	0.0110 (11)	0.0217 (10)	−0.0016 (9)	0.0009 (9)	0.0002 (9)
C14	0.0229 (12)	0.0108 (11)	0.0196 (10)	0.0013 (9)	0.0030 (9)	0.0018 (9)
C15	0.0369 (14)	0.0125 (12)	0.0295 (12)	−0.0012 (11)	−0.0089 (11)	0.0023 (10)
C16	0.0241 (12)	0.0163 (12)	0.0276 (12)	0.0004 (10)	−0.0049 (10)	0.0060 (10)
C17	0.0277 (13)	0.0115 (11)	0.0342 (13)	−0.0015 (10)	−0.0051 (10)	0.0025 (10)

### Geometric parameters (Å, º)

**Table:**

O1—C6	1.226 (3)	C7—H7A	0.9300
O2—C10	1.366 (3)	C8—C9	1.468 (3)
O2—C15	1.427 (3)	C8—H8A	0.9300
O3—C12	1.365 (3)	C9—C10	1.400 (3)
O3—C16	1.438 (3)	C9—C14	1.413 (3)
O4—C13	1.366 (3)	C10—C11	1.400 (3)
O4—C17	1.427 (3)	C11—C12	1.378 (3)
N1—C5	1.339 (3)	C11—H11A	0.9300
N1—C1	1.343 (3)	C12—C13	1.412 (3)
C1—C2	1.397 (3)	C13—C14	1.377 (3)
C1—C6	1.511 (3)	C14—H14A	0.9300
C2—C3	1.388 (4)	C15—H15A	0.9600
C2—H2A	0.9300	C15—H15B	0.9600
C3—C4	1.379 (4)	C15—H15C	0.9600
C3—H3A	0.9300	C16—H16A	0.9600
C4—C5	1.392 (3)	C16—H16B	0.9600
C4—H4A	0.9300	C16—H16C	0.9600
C5—H5A	0.9300	C17—H17A	0.9600
C6—C7	1.466 (3)	C17—H17B	0.9600
C7—C8	1.343 (3)	C17—H17C	0.9600
			
C10—O2—C15	117.81 (18)	O2—C10—C9	115.8 (2)
C12—O3—C16	117.48 (18)	C11—C10—C9	121.0 (2)
C13—O4—C17	117.05 (18)	C12—C11—C10	120.0 (2)
C5—N1—C1	117.3 (2)	C12—C11—H11A	120.0
N1—C1—C2	123.0 (2)	C10—C11—H11A	120.0
N1—C1—C6	117.17 (19)	O3—C12—C11	124.8 (2)
C2—C1—C6	119.9 (2)	O3—C12—C13	114.8 (2)
C3—C2—C1	118.6 (2)	C11—C12—C13	120.3 (2)
C3—C2—H2A	120.7	O4—C13—C14	125.5 (2)
C1—C2—H2A	120.7	O4—C13—C12	115.46 (19)
C4—C3—C2	118.9 (2)	C14—C13—C12	119.0 (2)
C4—C3—H3A	120.6	C13—C14—C9	122.0 (2)
C2—C3—H3A	120.6	C13—C14—H14A	119.0
C3—C4—C5	118.7 (2)	C9—C14—H14A	119.0
C3—C4—H4A	120.6	O2—C15—H15A	109.5
C5—C4—H4A	120.6	O2—C15—H15B	109.5
N1—C5—C4	123.5 (2)	H15A—C15—H15B	109.5
N1—C5—H5A	118.3	O2—C15—H15C	109.5
C4—C5—H5A	118.3	H15A—C15—H15C	109.5
O1—C6—C7	123.3 (2)	H15B—C15—H15C	109.5
O1—C6—C1	120.1 (2)	O3—C16—H16A	109.5
C7—C6—C1	116.6 (2)	O3—C16—H16B	109.5
C8—C7—C6	121.2 (2)	H16A—C16—H16B	109.5
C8—C7—H7A	119.4	O3—C16—H16C	109.5
C6—C7—H7A	119.4	H16A—C16—H16C	109.5
C7—C8—C9	126.9 (2)	H16B—C16—H16C	109.5
C7—C8—H8A	116.6	O4—C17—H17A	109.5
C9—C8—H8A	116.6	O4—C17—H17B	109.5
C10—C9—C14	117.6 (2)	H17A—C17—H17B	109.5
C10—C9—C8	120.0 (2)	O4—C17—H17C	109.5
C14—C9—C8	122.5 (2)	H17A—C17—H17C	109.5
O2—C10—C11	123.1 (2)	H17B—C17—H17C	109.5
			
C5—N1—C1—C2	0.3 (3)	C8—C9—C10—O2	0.9 (3)
C5—N1—C1—C6	−180.0 (2)	C14—C9—C10—C11	2.1 (3)
N1—C1—C2—C3	−0.8 (3)	C8—C9—C10—C11	−178.7 (2)
C6—C1—C2—C3	179.5 (2)	O2—C10—C11—C12	178.1 (2)
C1—C2—C3—C4	1.0 (3)	C9—C10—C11—C12	−2.4 (3)
C2—C3—C4—C5	−0.7 (3)	C16—O3—C12—C11	−2.8 (3)
C1—N1—C5—C4	0.0 (3)	C16—O3—C12—C13	177.9 (2)
C3—C4—C5—N1	0.2 (4)	C10—C11—C12—O3	−178.3 (2)
N1—C1—C6—O1	−177.7 (2)	C10—C11—C12—C13	1.0 (3)
C2—C1—C6—O1	2.0 (3)	C17—O4—C13—C14	−2.6 (3)
N1—C1—C6—C7	2.9 (3)	C17—O4—C13—C12	176.7 (2)
C2—C1—C6—C7	−177.4 (2)	O3—C12—C13—O4	0.7 (3)
O1—C6—C7—C8	−1.3 (4)	C11—C12—C13—O4	−178.7 (2)
C1—C6—C7—C8	178.0 (2)	O3—C12—C13—C14	−180.0 (2)
C6—C7—C8—C9	179.4 (2)	C11—C12—C13—C14	0.6 (3)
C7—C8—C9—C10	178.8 (2)	O4—C13—C14—C9	178.4 (2)
C7—C8—C9—C14	−2.0 (4)	C12—C13—C14—C9	−0.9 (3)
C15—O2—C10—C11	0.4 (3)	C10—C9—C14—C13	−0.4 (3)
C15—O2—C10—C9	−179.1 (2)	C8—C9—C14—C13	−179.7 (2)
C14—C9—C10—O2	−178.38 (19)		

### Hydrogen-bond geometry (Å, º)

**Table:**

*D*—H···*A*	*D*—H	H···*A*	*D*···*A*	*D*—H···*A*
C3—H3*A*···O4^i^	0.93	2.39	3.277 (3)	160
C15—H15*A*···N1^ii^	0.96	2.47	3.349 (3)	153

Symmetry codes: (i) *x*−1, −*y*+5/2, *z*−1/2; (ii) *x*, *y*−1, *z*.
